# Whole-Exome Sequencing Identified a Novel Compound Heterozygous Genotype in* ASL* in a Chinese Han Patient with Argininosuccinate Lyase Deficiency

**DOI:** 10.1155/2019/3530198

**Published:** 2019-04-30

**Authors:** Mei Zhao, Lingling Hou, Huajing Teng, Jinchen Li, Jiesi Wang, Kunlin Zhang, Lin Yang

**Affiliations:** ^1^Key Lab of Mental Health, Institute of Psychology, Chinese Academy of Sciences, 4A Datun Rd., Beijing 100101, China; ^2^Beijing Institutes of Life Science, Chinese Academy of Sciences, Beijing 100101, China; ^3^Department of Pediatrics, Second Affiliated Hospital of Xi'an Jiaotong University, Xi'an, Shaanxi Province, China

## Abstract

Pathogenic variants in the argininosuccinate lyase (*ASL*) gene have been shown to cause argininosuccinate lyase deficiency (ASLD); therefore, sequencing analysis offers advantages for prenatal testing and counseling in families afflicted with this condition. Here, we performed a genetic analysis of an ASLD patient and his family with an aim to offer available information for clinical diagnosis. The research subjects were a 23-month-old patient with a high plasma level of citrulline and his unaffected parents. Whole-exome sequencing identified potential related* ASL* gene mutations in this trio. Enzymatic activity was detected spectrophotometrically by a coupled assay using arginase and measuring urea production. We identified a novel nonsynonymous mutation (c.206A>G, p.Lys69Arg) and a stop mutation (c.637C>T, p.Arg213∗) in* ASL* in a Chinese Han patient with ASLD. The enzymatic activity of a p.Lys69Arg ASL construct in human embryonic kidney 293T cells was significantly reduced compared to that of the wild-type construct, and no significant activity was observed for the p.Arg213∗ construct. Compound heterozygous p.Lys69Arg and p.Arg213∗ mutations that resulted in reduced ASL enzyme activity were found in a patient with ASLD. This finding expands the clinical spectrum of ASL pathogenic variants.

## 1. Introduction

Argininosuccinate lyase deficiency (ASLD), which is also called argininosuccinic aciduria (ASA), is the second most common urea-cycle disorder, with an estimated incidence of 1:70,000 live births in the USA [1]. ASLD is caused by reduced or absent activity of argininosuccinate lyase (ASL), which catalyzes the hydrolytic cleavage of ASA into arginine and fumarate [2, 3]. The* ASL* gene is located on chromosome 7q11.21 and approximately 17 kb in length and contains 17 exons [3]. Over 150 variants of the* ASL* gene have been deposited in the Leiden Open Variation Database and Human Gene Mutation Database [1]. To our knowledge, two Chinese patients with neonatal onset ASLD have been described that have compound heterozygous ASL mutations, c.434A>G/c.1336C>T and c.544C>T/c.706>C.T [4, 5].

The clinical manifestations of ASLD are highly heterogeneous [6]. ASLD can be divided into two common types according to the time at onset: severe neonatal onset and late onset [7]. Patients with neonatal onset are typically diagnosed with life-threatening hyperammonemia within the first few days after birth [8]. In patients with the late onset type, ASLD is triggered by acute infection after the newborn period, which results in neurological and intellectual impairment [6]. Currently, a diagnosis of ASLD is established by the presence of elevated ASA and citrulline levels and reduced ASL enzyme activity in urine or blood, which are measured by biochemical testing and enzymatic activity testing, respectively. Although these tests are sufficient to diagnose almost all patients, molecular genetic testing is still useful for family counseling and prenatal testing [7]. However, there are few reports on the associations between the* ASL* gene variant and enzyme activity. In this study, whole-exome sequencing was performed on a family trio to identify the causative mutation(s). In addition, the influence of the mutations on enzyme activity was evaluated by using mutant ASL constructs transfected into human embryonic kidney 293T cells. This study aimed at offering more information for the genetic detection of ASLD.

## 2. Materials and Methods

### 2.1. Case Presentation

Subjects from a parent-offspring trio were collected. The proband was a 23-month-old male Chinese child who presented four episodes of tonic seizures over 10 days at the age of 16 months, with normal parents. The patient had enamel hypoplasia and hypotonia of the trunk and limb muscles. His developmental quotient (DQ) and mental index (MI) scores were both less than 70. The brain MRI revealed long T1 and long T2 signal changes in the white matter adjacent to the anterior and posterior horns of both lateral ventricles, indicating dysplasia in these regions. Video-electroencephalography (EEG) monitoring showed abnormal brain waves during sleep. Increased plasma alanine aminotransferase (ALT) and aspartate aminotransferase (AST) were noted in liver function tests (ALT: 117 IU/L, normal range: 9–50 IU/L; AST: 50 IU/L, normal range: 15–40 IU/L). Plasma bilirubin was in the normal range. Elevated plasma citrulline (220.94 *μ*mol/L, normal range: 7.00–35.00 *μ*mol/L) and normal plasma arginine were also noted. Seven months later, his plasma ammonia level was increased to 175 *μ*mol/L (normal range: 11–51 *μ*mol/L) and his plasma levels of ALT (335 IU/L), AST (106 IU/L), and citrulline were higher than before. The brain MRI showed ventricular dilation and cerebral atrophy of the left hemisphere with malacia, suggesting serious irreversible brain injury. After 2 weeks of treatment with a restricted protein diet and arginine supplementation, his plasma ammonia level was decreased to 70 *μ*mol/L. However, the patient died of a serious infection several months after hospital discharge.

Informed written consent was obtained from all participants, and the genetic analysis of the infant was performed with written consent from his parents. The study was approved by the review board of the Institute of Psychology, Chinese Academy.

### 2.2. Whole-Exome Sequencing

Genomic DNA was extracted from peripheral blood lymphocytes and analyzed by gel electrophoresis to confirm its high-molecular weight. Exome capture and library preparation were performed using the Agilent SureSelect Human All Exon V4 kit according to the manufacturer's protocols. In brief, 3 *μ*g of DNA from each of the three individuals was sheared to about 200 bp using adaptive focused acoustic technology. Then the sheared samples were end repaired, A-tailed, and ligated with the SureSelect Adaptor Oligo Mix. After ligation, the adaptor-ligated libraries were amplified by PCR and validated with an Agilent 2100 Bioanalyzer before capture. In-solution hybridization was performed at 65°C for 24 hours by combining the precapture DNA library with hybridization buffers, blocking reagents, and the SureSelect biotinylated capture library, according to Agilent's standard protocol. Then, the captured targets were isolated with streptavidin-coated beads and multiplexed by posthybridization amplification. The final enriched libraries were analyzed on the HiSeq 2000 platform (Illumina) by sequencing of paired-end 100 bp reads.

### 2.3. Read Mapping and Variant Analysis

The raw reads were firstly trimmed for low quality bases and adaptor sequences with Trimmomatic [9]. Then, the paired reads were mapped to the reference human genome (hg19) using the Burrows-Wheeler Aligner (BWA 0.7.8). After alignment, duplicated reads were marked with Picard [10]. Indel realignment and base quality recalibration were carried out using Genome Analysis Toolkit (GATK 3.2.2) [11]. Variants were called individually in each sample using GATK Haplotype Caller within 100 bp upstream and downstream of the capture targets and written in genomic variant call format (GVCF). A joint genotyping analysis was performed on the GVCF files produced for the family trio and was written in variant call format (VCF). Then, the variants with a minimum read depth of 10 were filtered against dbSNP (build 138), the 1000 Genome Project, and EPS6500. Subsequently, the VCF file was annotated using SnpEff [12] and loaded into GEMINI [13] for further analysis. Three built-in analysis tools of GEMINI, de novo, autosomal recessive, and compound heterozygote, were used to identify the disease-causing mutation.

### 2.4. Structural Analysis of the ASL Protein

The impact of the K69R substitution on the function of ASL was predicted by using SIFT and PolyPhen-2 software. For the structural analysis, the human argininosuccinate lyase tetramer model was built by using SWISS-MODEL (https://swissmodel.expasy.org/) with the best-matched template (PDB (http://www.rcsb.org) entity: 1K62). Then, we added the L-arginine ligands to the human argininosuccinate lyase tetramer model by aligning the crystal structure of* Thermus thermophilus* argininosuccinate lyase complex (PDB entity: 2E9F) with the model. The structure alignment and visualization were performed by using Chimera.

### 2.5. Sanger Sequencing

Candidate mutations were verified by PCR and Sanger sequencing on an ABI3730 sequencer (Beijing Genomics Institute) using the following primers: the primer pair for the c.206A>G-containing allele (forward, GGAGGGTGGAGGAGAGACTA and reverse, GCAGTCATCAAGGCACACAG) and the primer pair for the c.637C>T-containing allele (forward, CCAGGGAAGAGGCTAAGCG and reverse, CCATGAGTGACCCCTGAACT).

### 2.6. Expression of ASL Constructs Transfected into Human Embryonic Kidney 293T Cells

Full-length wild-type ASL cDNA (1395 bp) and the p. Lys69Arg and p.Arg213Ter mutant ASL cDNAs were synthesized by Genewiz (Suzhou, China). The cDNAs were cloned into the pcDNA3.1 vector (Invitrogen) with a c-Myc tag. All constructs were validated by sequencing as described above in the Sanger sequencing section.

Human embryonic kidney 293T cells were chosen for the experiment due to their low background ASL activity [14]. The 293T cells were cultured in Dulbecco's modified Eagle's medium (DMEM; Gibco, Grand Island, NY, USA) supplemented with 10% fetal bovine serum (FBS) and 1% PS (Gibco) overnight at 37°C in 5% CO_2_. Then, the cells were separated equally into 4 wells (~2 × 10^5^ cells per well) and transfected with 7 *μ*g of the p-wt, p.Lys69Arg, and p.Arg213Ter constructs using Lipofectamine™ 3000 (Life Technologies, Carlsbad, CA, USA) transfection regent. The empty vector (EV) pcDNA3.1 was included as a negative control. The cells were harvested 48 h after transfection.

### 2.7. ASL Enzyme Activity Assay in Transfected 293T Cells

ASL activity was detected by a coupled assay using arginase and measuring urea production as described previously [15]. First, 50 *μ*l of cell extract was mixed with 100 *μ*l of 13.6 mM argininosuccinate in water and 100 *μ*l of arginase (50 units) (Sigma-Aldrich, St. Louis, MO, USA) in 66.7 mM PBS (pH 7.5). The reactions were stopped after 30 min by adding perchloric acid (2% final concentration). Then, a 10-*μ*l aliquot of the stopped reaction was sampled to assay the urea concentration with a urea assay kit (Sigma-Aldrich). The ASL activities of the wild-type and mutant proteins were expressed as the concentration of urea/the total protein concentration in mU/mg protein. The total protein concentration was assayed by using the Bicinchoninic acid protein assay kit (CWBIO, Beijing, China). All experiments were performed in triplicate.

### 2.8. Data Analysis

ASL activity was analyzed by one-way analysis of variance (ANOVA) followed by Tukey's multiple comparison test by using GraphPad Prism 6 software. For all data analyses, statistical significance was set at p values less than 0.05.

## 3. Results

### 3.1. Whole-Exome Sequencing Analysis

**Figure 1:**
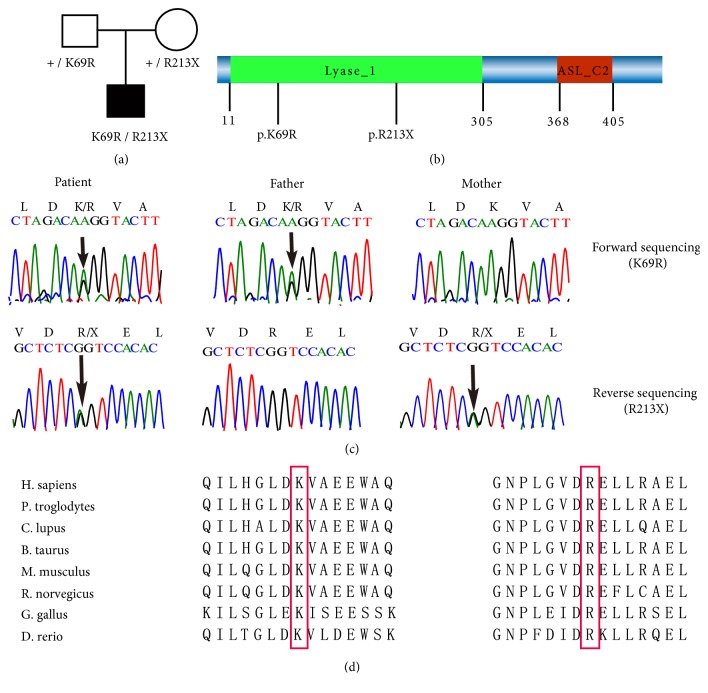
Identification of the* ASL* mutations in the patient with ASLD and his parents. (a) Pedigree of the family with ASLD. Males are represented by squares, females are represented by circles, and affected members are indicated by filled symbols. (b) Schematic diagram of the full-length ASL protein. The p.Lys69Arg mutation is located in exon 3, whereas p.Arg213Ter is in exon 9. (c) Both the c.206A>G (p.Lys69Arg) and c.637C>T (p.Arg213Ter) mutations in* ASL* were confirmed in the patient and his parents by Sanger sequencing. (d) Lys 69 is evolutionarily conserved.

Approximately 91% of all sequenced bases had a Phred quality score above Q30. On average, each sample generated a total of ~5.4 Gb of data, and >99% was successfully aligned to the human reference genome (Table 1). The target regions were, on average, covered ~65×, and 97% of these had a minimum coverage depth of 20×. The variant call was performed with GATK Haplotypecaller, and more than 70,000 variants were called in each sample, with a minimum read depth of 10×. Then, we filtered the variants against dbSNP (build 138), the 1000 Genome Project, EPS6500, ExAC, and an in-house database of 1,500 Chinese exomes, with an allele frequency >0.0001 [16]. After filtering, the built-in analysis tool in GEMINI was used to identify the disease-causing mutations using three models:* de novo*, autosomal recessive, and compound heterozygotes. A total of nine different genes (18 variants) were identified by GEMINI with a predicted impact severity of “high” or “medium,” including three in the autosomal recessive model (ACE2, FAM20C, and HLA-DRB1) and six in the compound heterozygotes model (NIPA1, ASL, MYO18A, SPATC1, PTPN22, and RASSF1). None were identified in the* de novo* model. The variants were manually filtered by known disease association and clinical phenotype similarity and finally prioritized to the candidate gene,* ASL*. Two variants of* ASL*, including one novel nonsynonymous mutation (c.206A>G, p.Lys69Arg) and one stop gain mutation (c.637C>T, p.Arg213∗), were identified. These variants were predicted to cause argininosuccinate synthetase deficiency. Sanger sequencing confirmed that the affected child inherited the missense mutation from his father and the stop gain mutation from his mother (Figure 1).

### 3.2. Structural Analysis of the ASL Protein

**Figure 2:**
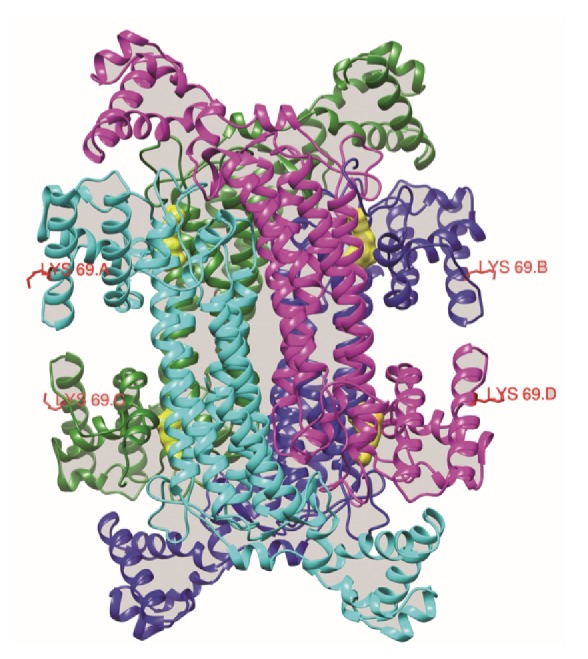
Structure of the ASL protein. Red indicates a spiral, yellow indicates a slice, and blue indicates a corner.

Although SIFT and PolyPhen-2 analyses did not detect the p.Lys69Arg residue as deleterious, 3D structural analysis revealed that the residue Lys was located at the surface of the ASL protein near the active site for arginine (Figure 2). In contrast, residue Arg213 was located near to the active center of protein. Therefore, these alterations were predicted to affect protein function.

### 3.3. Enzymatic Activities of ASL Constructs Transfected in 293T Cells

**Figure 3:**
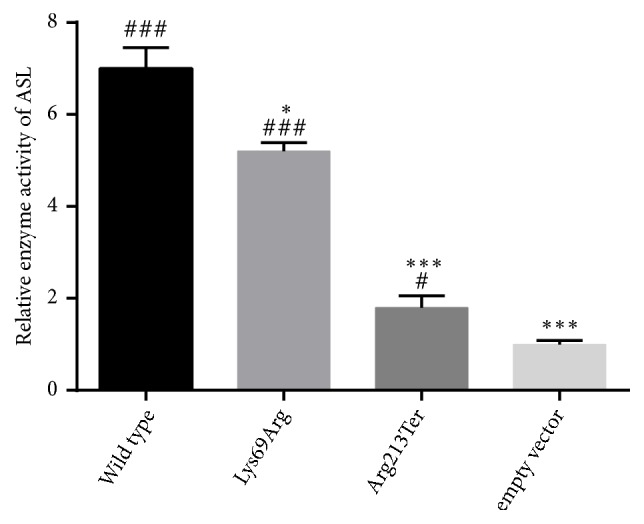
Effects of the* ASL* mutations on enzymatic activity. The enzyme activities of the* ASL* mutants in (co)transfected 293T cell extracts are significantly reduced compared to the wild-type-transfected cells. ^*∗∗∗*^*p* < 0.001 and ^*∗*^*p* < 0.05 versus wild type; ^###^*p* < 0.001 and ^#^*p* < 0.05 versus empty vector.

The results of the initial ASL enzymatic activity experiment showed remarkably reduced activity in the empty vector-transfected cells when compared to the activity of the wild-type ASL-transfected cells (*p *< 0.001), indicating that the ASL construct-transfected cells showed the expected difference in ASL activity and thus were suitable for the assay. Cells transfected with the p.Lys69Arg mutant displayed reduced enzymatic activity compared to the wild-type ASL-transfected cells (*p *< 0.05). In addition, the enzymatic activity in the p.Arg213∗ mutant-transfected cells was significantly decreased compared to the levels in the wild-type ASL-transfected cells (*p *< 0.001, Figure 3).

## 4. Discussion

ASLD is usually recessively inherited, and a series of ASL mutations that are related to the variable phenotypic expression of ASLD have been reported [1]. However, there is a relatively low prevalence of ASLD in East Asian populations, and only a small number of mutations related to urea-cycle defects have been reported [17]. Whole-exome sequencing is considered to be a reliable method for the diagnosis of gene mutations in metabolic diseases [18]. In the present study, mutation screening of a family trio affected by ASLD was performed by using whole exon sequencing, and two novels variants, p.Lys69Arg and p.Arg213Ter, in the* ASL* gene were detected.

To date, over 300 mutations scattered throughout the* ASL* gene have been detected in ASLD patients [19]. A previous study suggested that four novel mutations (p.E189G, p.R168C, p.R126P, and p.D423H) are responsible for the low ASL activities in Austria patients with ASLD [20]. In addition, Tanaka* et al.* detected a new homozygous missense mutation, 1395G>C, in* ASL* that can lead to termination at amino acid position 465 (X465Y) [21]. Reportedly, most mutations in ASLD patients are located in a mutational hotspot of* ASL* (exon 7) [22]. However, the compound heterozygous mutations p.Lys69Arg and p.Arg213Ter found in the present study are located in exons 3 and 9, respectively. In addition, c.206A> G located at the second last nucleotide of the exon 3 of ASL gene and could affect the splicing site of exon 3. This could explain the individual susceptibility of these mutations in mutational events and expand the clinical spectrum of ASL variants.

Previous studies revealed that complementing alleles at the* ASL* locus play an important role in the restoration of ASL activity [23, 24]. Thus, homozygous or heterozygosis variants in different regions are responsible for the reduced ASL activity in ASDL patients. Linnebank* et al.* showed compound heterozygosity, with 257A>C in exon 3 and 337C>T in exon 4, in the* ASL* gene in patients with neonatal onset ASLD [25]. In this study, we identified compound heterozygous mutation in* ASL* in a Chinese patient with the late onset form of ASLD. The compound heterozygous mutation in the* ASL* gene was composed of a novel nonsynonymous mutation (c.206A>G, p.Lys69Arg) and a known stop gain mutation (c.637C>T, p.Arg213Ter). Three-dimensional structural analysis revealed that the Lys residue was located at the surface of the ASL protein near the active site for arginine, which might affect the function of ASL. The other mutation resulted in a stop codon at amino acid position 213, which was located near the active center of protein. Early termination of translation could affect the structure and stability of the ASL protein, leading to reduced ASL enzyme activity.

There is limited evidence showing the correlation between specific mutations and particular clinical courses [26, 27]. The correlation between ASL enzyme activity and clinical outcomes has been evaluated by using different expression systems and cell types, including recombinant purified ASL protein from prokaryotic and eukaryotic systems and yeast or human embryonic kidney 293T cell lysates [14]. In this study, we measured the ASL activity of individual alleles in 293T cells. Our results showed that the mutant p.Lys69Arg protein displayed reduced enzymatic activity compared to the wild-type protein, whereas the p.Arg213Ter mutant displayed no activity. Interestingly, two acetylated residues in ASL, Lys69 and Lys288, have been identified and acetylation of ectopically expressed ASL has been demonstrated. In most intermediate metabolic enzymes that are acetylated, the acetylation can directly affect enzyme activity or stability, and the Lys to Arg mutant in ASL reduces enzymatic activity by changing the acetylation state of ASL [28]. Our study provided strong support for the effect of acetylation on ASL activity by examining the Lys69Arg mutation identified in an ASLD patient. Therefore, the p.Lys69Arg mutation in a compound heterozygous state along with a stop mutation was the cause of late onset ASLD in our patient due to reduced ASL enzyme activity.

The variant (c.206A>G, p.Lys69Arg) was analyzed to affect the splicing site of exon 3 of ASL gene and had been detected to be a pathogenic variant; thus it was necessary to measure mRNA transcripts and mRNA stability of ASL by RT-PCR amplification. However, due to the fact that this hypothesis was just put forwarded after the premature death of the patient, it was a pity that we failed to verify the enzyme deficiency whether was caused by an aberrant splicing of exon 3. In our further study, we hope to find a new way for evaluating this hypothesis and confirm it.

## 5. Conclusions

In this study, whole-exome sequencing revealed a novel nonsynonymous mutation (c.206A>G, p.Lys69Arg) and a known stop gain mutation (c.637C>T, p.Arg213Ter) in* ASL* in a Chinese Han patient with ASLD. This compound heterozygous mutation resulted in reduced ASL enzyme activity. Our study expands the clinical spectrum of known ASL mutations.

## Figures and Tables

**Table 1:** Summary of the whole exome sequencing data generated in this study.

	Patient	Father	Mother

Total reads	66474926	33052118	61697322
Reads mapped on genome	65898400	32947126	61498982
Reads mapped on genome (%)	99.1	99.7	99.7
Bases mapped on target	4025794210	2006750456	3751368029
Bases mapped on target (%)	60.6	60.8	61.0
Average depth on target	79.9	39.8	74.4
Bases on target with depth ≥20×	3991310493	1869091335	3712704877
Bases on target with depth ≥20× (%)	99.14	93.14	98.97
Total number of SNVs	76050	61169	75072
Total number of INDELs	11597	8695	11394
Total number of Ti	53422	43323	52735
Total number of Tv	22628	17846	22337
Homozygous variants	36085	28288	35426
Heterozygous variants	51562	41576	51040

∗Variants with depth ≥10 are presented; ∗ multiallelic sites are excluded.

## Data Availability

The data used to support the findings of this study are available from the corresponding author upon request.
